# Awareness of Keratoconus Among the Population of Taif City, Saudi Arabia: A Cross-Sectional Study

**DOI:** 10.7759/cureus.72170

**Published:** 2024-10-22

**Authors:** Abdulaziz M Alshehri, Mashael Bajunaid, Rahaf A Althobaiti, Hanouf Althobaiti, Abdulmajeed Alkhathami, Azza A Taha

**Affiliations:** 1 Surgery, Taif University, Taif, SAU; 2 College of Medicine, Taif University, Taif, SAU; 3 College of Medicine and Surgery, Taif University, Taif, SAU; 4 Collage of Medicine, Taif University, Taif, SAU; 5 Ophthalmology, King Fahad Hospital, Al Baha Health Cluster, Al Baha, SAU; 6 Family and Community Medicine, Taif University, Taif, SAU

**Keywords:** awareness, corneal thinning, eye rubbing, keratoconus, saudi arabia, taif, visual impairment

## Abstract

Background: Keratoconus (KC) is a prevalent corneal condition in Saudi Arabia, with studies suggesting variable prevalence rates across regions, highlighting a considerable public health issue. Despite its prevalence, public awareness of the condition remains low. This study aims to evaluate the level of awareness of keratoconus among the population in Taif City, Saudi Arabia.

Methods: A community-based cross-sectional study was conducted in Taif City from May to July 2024. An online questionnaire, adapted from a validated Arabic version, was distributed via social media to adults aged 18 years and older. The survey included questions about demographics, medical history, and keratoconus awareness. Data were analyzed using descriptive and inferential statistics. Awareness levels were categorized as good or poor based on participants' responses, and the chi-squared test or Fisher’s exact test was used to compare categorical variables.

Results: The study included 499 participants, with 282 (56.5%) aged 18-25 years and 383 (76.8%) females. Nearly half of the participants, 248 (49.7%), had a history of allergies, and 294 (58.9%) reported having an eye condition. While 286 (57.3%) had heard of keratoconus, only 83 (16.6%) correctly identified it as corneal thinning. Additionally, 263 (52.7%) believed frequent eye rubbing could lead to keratoconus, though 186 (37.3%) were unsure. Overall, 414 (82.9%) participants demonstrated poor awareness of keratoconus. Participants with a refractive error, a family history of KC, or prior knowledge of the disease were significantly more likely to have good awareness (p < 0.05).

Conclusion: The study reveals a low level of awareness about keratoconus among the population in Taif, despite its significant association with eye rubbing and potential visual impairment. The findings highlight the need for public health interventions and educational campaigns to raise awareness and promote early detection of keratoconus.

## Introduction

Keratoconus (KC) is a progressive, bilateral, non-inflammatory corneal condition marked by thinning and conical deformation, leading to irregular astigmatism and vision impairment [1]. It typically emerges in individuals during their twenties or thirties, progressing into their forties [1,2]. The disease's pathophysiology involves the breakdown of corneal collagen, compromising its integrity and deteriorating vision [1]. The prevalence varies globally, with some regions, such as the Ural region in Russia, reporting rates as high as 6.45% [3], while parts of the Middle East, including Iran, indicate a 3-4% prevalence among the elderly [4]. These regional differences are attributed to genetic and environmental factors, including UV exposure and ocular allergies [3,4].

Early detection is key to managing KC; however, public awareness is often insufficient, delaying diagnosis and treatment [5]. Lack of awareness about risk factors and symptoms can lead to late-stage diagnoses when corneal damage is advanced, though early intervention, like corneal collagen cross-linking, can slow disease progression [6]. Awareness efforts are further complicated by varying diagnostic criteria, which make prevalence comparisons difficult [7].

Keratoconus (KC) prevalence and awareness have gained significant attention in Saudi Arabia due to its relatively high occurrence. A study in the Eastern Province found a 2.75% prevalence among individuals aged 13-23, with 11.93% showing subclinical keratoconus (SKC), and eye rubbing, a known risk factor, was more frequent in KC patients (33.3%) [8]. In Taif, 8.59% of laser vision correction patients were diagnosed with KC, with 6.55% having bilateral KC and 9.46% exhibiting SKC [9]. Familial predisposition and environmental factors, such as UV exposure, are believed to contribute to the regional prevalence [9]. Despite the high incidence, awareness remains low; in the Aseer region, 85.74% of participants had inadequate knowledge, while 74% of those in the Eastern Province showed limited understanding [8,10]. Additionally, 81.4% of respondents in the latter region reported frequent eye rubbing due to allergies or other factors, further highlighting the need for better public education and early intervention efforts [10].

Given the high prevalence and low awareness in Saudi Arabia, this study seeks to evaluate KC awareness and its relationship to eye rubbing in Taif City, Saudi Arabia, aiming to highlight the need for targeted education and early intervention.

## Materials and methods

Study design and participants

This community-based cross-sectional study was performed in Taif City, Saudi Arabia, focusing on individuals aged 18 years and above. Data gathering occurred from May 2024 until July 2024. Participants were recruited via the distribution of an online questionnaire, available in Arabic, and circulated across multiple social media channels. The recruitment method sought to involve a diverse portion of the population to guarantee a representative sample. 

Sample size calculation

The minimum required sample size for the study was determined utilizing the Raosoft sample size calculator with the parameters of a population percentage of 50%, a margin of error of 5%, and a confidence interval (CI) of 95%. Utilizing the equation: n = (Z² * p * (1 - p)) / e². The minimum required sample size was calculated to be 384 people, where Z is 1.96 (indicating a 95% confidence level), p is 0.5 (the presumed population proportion), and e is 0.05 (the margin of error). We aimed to recruit a minimum of 450 participants for this study to guarantee the reliability of the results and to account for potential withdrawals and non-responses. A total of 499 participants completed the survey.

Questionnaire development and distribution

The questionnaire used in this study was adapted from a previously validated Arabic version developed by Kordi et al. [11]. It consisted of three main sections. The first section gathered sociodemographic information, including age, gender, education level, and income. The second section focused on participants' medical history, specifically regarding allergies, eye diseases, and vision problems, using a series of yes-or-no questions. The third section evaluated participants' awareness of keratoconus through nine questions covering knowledge of the disease, its causes, and available treatment options. To guarantee widespread dissemination across diverse demographic and geographic groups in Taif City, the survey was disseminated through a variety of social media platforms, such as Meta (formerly Facebook [California, USA]), X (formerly Twitter [California, USA]), and WhatsApp (California, USA).

Informed consent and ethical approval

All participants were provided with a comprehensive explanation of the study's objectives before their participation, and informed consent was obtained. Participation was entirely voluntary, and confidentiality was assured throughout the process. The study protocol received permission from the Deanship of Scientific Research at Taif University (approval number: KFU-REC-2023-FEB-ETHICS585) on February 15, 2023.

Conflict of interest

The authors do not have any conflict of interest.

Data analysis

Descriptive and inferential statistical methods were implemented to analyze the data. Categorical variables were presented as frequencies and percentages, while continuous variables were reported as means with standard deviations. A scoring system was implemented to evaluate the level of awareness of keratoconus among participants, with each correct response contributing to the aggregate awareness score. Participants who achieved a score of 75% or higher were classified as having good awareness, while those who scored below 75% were considered to have inadequate awareness. The chi-squared test or Fisher's exact test was employed to compare categorical variables between groups, as applicable. Statistical significance was defined as a p-value of less than 0.05. IBM Corp. Released 2020. IBM SPSS Statistics for Windows, Version 27.0. Armonk, NY: IBM Corp. was employed to conduct all analyses. In addition, Python was used to generate visualizations of key findings.

## Results

Sociodemographic characteristics

The study included a total of 499 participants. The majority, 282 (56.5%), were aged between 18 and 25 years, followed by 74 (14.8%) aged 36 to 45 years, 67 (13.4%) aged 26 to 35 years, and 76 (15.2%) aged older than 45 years. In terms of gender distribution, 383 (76.8%) were female, while 116 (23.2%) were males. Regarding education, 325 participants (65.1%) had completed university or higher education, and 130 (26.1%) had completed secondary education. A total of 262 participants (52.5%) reported having a monthly income of less than 3,000 Saudi Riyals (SR), 134 (26.9%) earned between 3,000 and 10,000 SR, and 86 (17.2%) reported earning more than 10,000 SR. Most participants, 470 (94.2%), resided in the Western region of Saudi Arabia (Table 1).

**Table 1:** Sociodemographic data of participants in the study

Parameter	Level	Number of subjects	Percentage, %
Age (years)	18 to 25	282	56.5
	26 to 35	67	13.4
	36 to 45	74	14.8
	Older than 45	76	15.2
Gender	Males	116	23.2
	Females	383	76.8
Education level	Illiterate	3	0.6
	Elementary	13	2.6
	Intermediate	28	5.6
	Secondary	130	26.1
	University/Higher Education	325	65.1
Income, SR	<3000	262	52.5
	3000 to 10000	134	26.9
	>10000	86	17.2
Place of residence	Western	470	94.2
	Central	13	2.6
	Southern	12	2.4
	Northern	3	0.6
	Eastern	1	0.2

History of allergies and eye conditions

Nearly half of the participants, 248 (49.7%), reported a history of allergies, with the most common type being eye allergies, affecting 151 participants (54.3%). This was followed by skin allergies in 105 participants (21.0%) and chest allergies in 93 participants (18.6%). In terms of eye conditions, 294 participants (58.9%) reported having a refractive error or another eye condition. Among these, myopia or hyperopia was the most common condition, affecting 204 participants (40.9%). A small number of participants, 22 (4.4%), had been diagnosed with keratoconus, and 90 (18.0%) reported a family history of the disease. Additionally, 73 participants (14.6%) used contact lenses, and 14 participants (2.8%) had previously undergone eye surgery (Table 2).

**Table 2:** Allergic and eye disease history among participants in the study

Parameter	Level	Count (N)	Percentage, %
Had a history of allergies?	Yes	287	49.7
	No	251	50.3
Type of allergy	Skin allergy	105	21.0
	GIT allergy	52	10.4
	Eye allergy	151	54.3
	Chest allergy	93	18.6
	Nasal allergy	13	2.6
	Food and / or antibiotics allergy	1	0.2
Had a refractive error or any other eye condition?	Yes	294	58.9
	No	205	41.1
What is the eye disorder?	Myopia/hyperopia	204	40.9
	Previous refractive surgery	38	7.6
	Keratoconus	22	4.4
	Astigmatism	10	2.0
	Previous eye surgery	14	2.8
	Using contact lenses	73	14.6
	Others	8	1.6
Family history of keratoconus	Yes	90	18.0
	No	409	82.0

Awareness and perception of keratoconus

**Figure 1:**
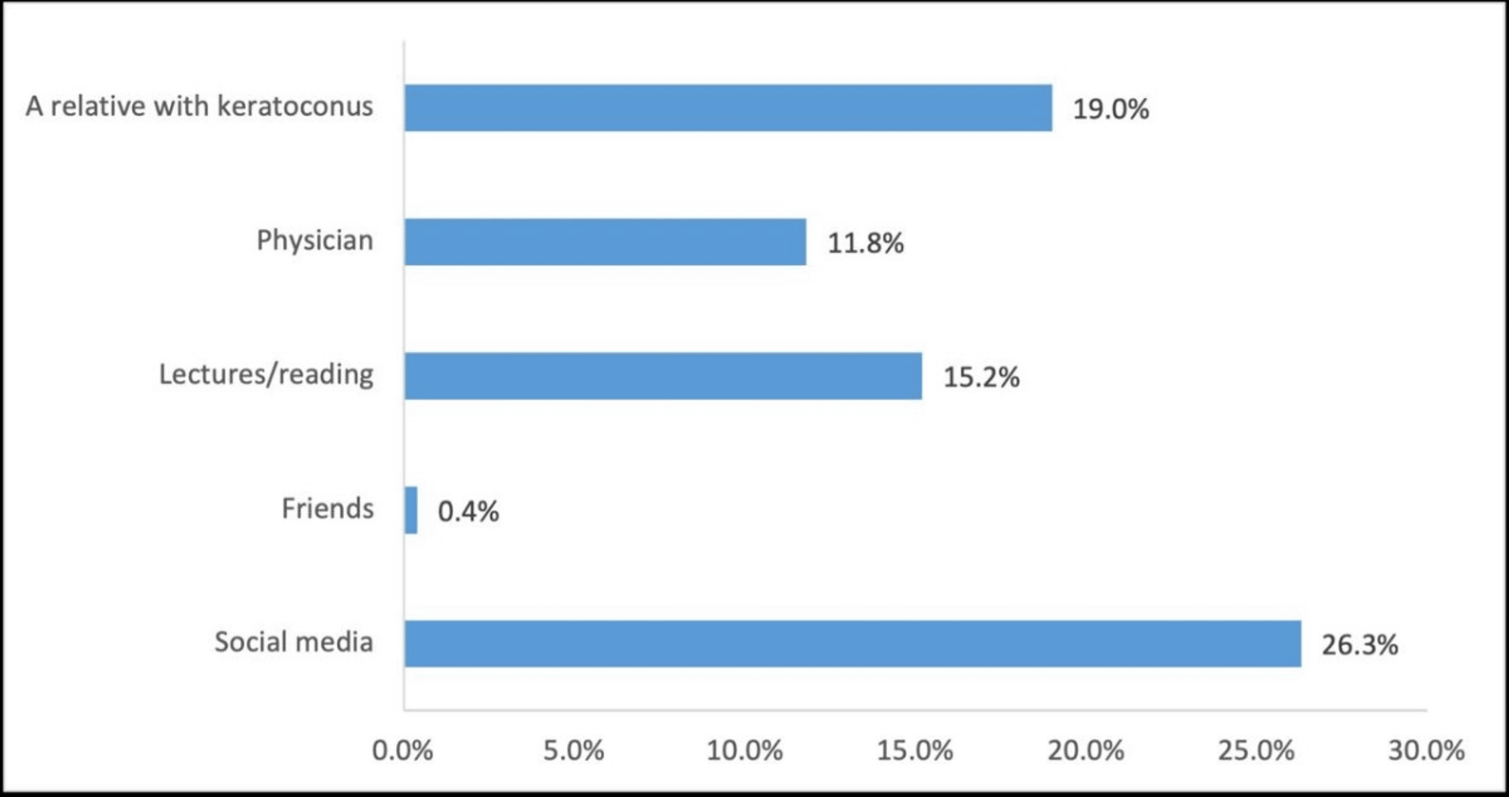
Source of information about keratoconus

A total of 286 participants (57.3%) had heard of keratoconus, with social media being the most common source of information for 131 participants (26.3%), followed by 95 participants (19.0%) who learned about keratoconus from relatives with the condition (Figure 1).

However, only 83 participants (16.6%) correctly identified keratoconus as corneal thinning, while 197 (39.5%) stated that they did not know what keratoconus was. Furthermore, 202 participants (40.5%) believed there was a relationship between keratoconus and allergies. Regarding the impact of keratoconus on vision, 313 participants (62.7%) recognized that it could lead to visual impairment. The most commonly identified treatment modality was surgery, cited by 237 participants (47.5%), although 151 participants (30.3%) were unsure about treatment options. Regarding eye rubbing, more than half of the participants, 263 (52.7%), believed that frequent eye rubbing could lead to keratoconus, while 186 participants (37.3%) were uncertain about this association (Table 3).

**Table 3:** Awareness and perception of keratoconus and eye rubbing among participants in the study

Parameter	Level	Count (N)	Percentage, %
Heard about keratoconus	Yes	286	57.3
	No	223	44.7
Source of information	Social media	131	26.3
	Friends	2	0.4
	Lectures/reading	76	15.2
	Physician	59	11.8
	A relative with keratoconus	95	19.0
What is keratoconus?	Thinning of corneal thickness	83	16.6
	Increased corneal thickness	97	19.4
	Immune disease	33	6.6
	Inflammation of the cornea	89	17.8
	I don’t know	197	39.5
Is there a relationship between keratoconus and allergy?	Yes	202	40.5
	No	64	12.8
Does keratoconus lead to visual impairment?	Yes	313	62.7
	No	34	6.8
	I don’t know	152	30.5
What are the treatment modalities of keratoconus?	Surgery	237	47.5
	Medical contact lenses	80	16.0
	Eyedrops	120	24.0
	Prescription glasses	113	22.6
	There are no treatments	19	3.8
	I do not know	151	30.3
Frequent eye rubbing habit and keratoconus	A habit that may lead to keratoconus	263	52.7
	It is a safe habit	50	10.0
	I don’t know	186	37.3

Overall awareness of keratoconus

**Figure 2:**
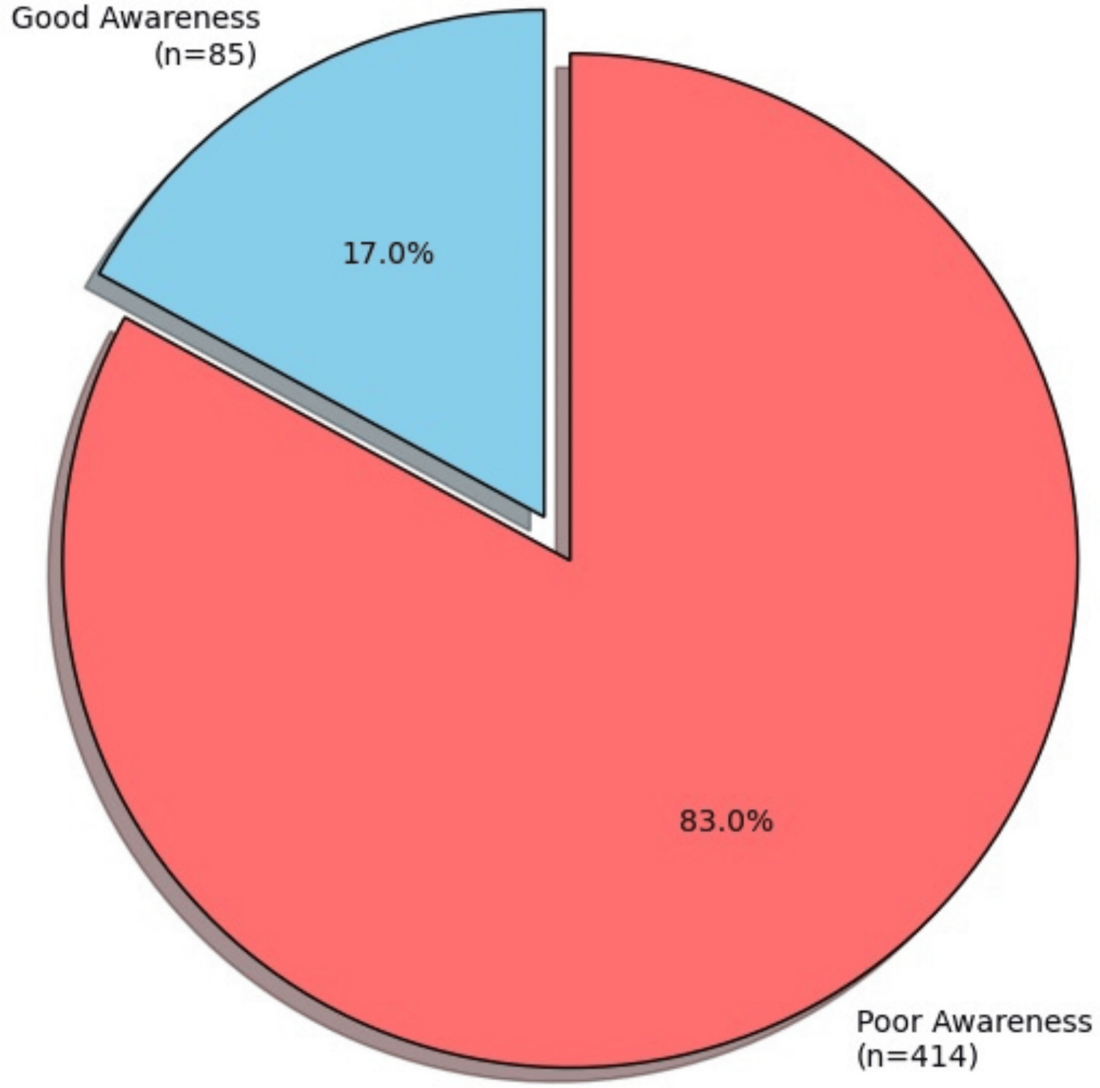
Overall awareness of keratoconus

The overall awareness of keratoconus was categorized as good for 85 participants (17.1%), while 414 participants (82.9%) demonstrated poor awareness (Figure 2). 

There was no significant difference in awareness between males, with 16 participants (13.8%) showing good awareness, and females, with 69 participants (18.0%) showing good awareness (p=0.326). Most males and females exhibited poor awareness of keratoconus in similar percentages, with notably higher participation from females compared to males. Awareness was also not significantly associated with age (p=0.262) or education level (p=0.599). However, participants with a refractive error or other eye conditions had significantly better awareness, with 61 participants (20.7%) demonstrating good awareness compared to 24 participants (11.7%) without such conditions (p=0.008). Additionally, those who had heard about keratoconus were significantly more likely to have good awareness, with 80 participants (28.0%) demonstrating good awareness compared to 5 participants (2.3%) who had not heard of the disease (p<0.001). Participants with a family history of keratoconus were also more likely to have good awareness, with 26 participants (28.9%) demonstrating good awareness compared to 59 participants (14.4%) without a family history (p=0.002) (Table 4).

**Table 4:** Overall awareness of the participants in the study as to keratoconus

Parameter	Level	Good awareness		Poor awareness		p-value
		n	%	n	%	
Gender	Males	16	13.8	100	86.2	0.326
	Females	69	18.0	314	82.0	
Age	18-25	54	19.1	228	80.9	0.262
	26-35	13	19.4	54	80.6	
	36-45	8	10.8	66	89.2	
	Older than 45	10	13.2	66	86.8	
Education level	Illiterate	0	0	3	100	0.599
	Elementary	1	7.7	12	92.3	
	Intermediate	7	25.0	21	75.0	
	Secondary	21	16.2	109	83.8	
	University/higher education	56	17.2	269	82.8	
Had a refractive error or any condition that affects the eye	Yes	61	20.7	233	79.3	0.008
	No	24	11.7	181	88.3	
Heard about keratoconus	Yes	80	28.0	206	72.0	<0.001
	No	5	2.3	208	97.7	
Family history of keratoconus	Yes	26	28.9	64	71.1	0.002
	No	59	14.4	350	85.6	

## Discussion

This study aimed to evaluate the level of awareness about keratoconus (KC) among the population of Taif City, Saudi Arabia. The findings revealed that, although more than half of the participants had heard of keratoconus, the majority demonstrated poor awareness regarding the disease's specific characteristics, causes, and treatment options. This substantial gap in knowledge is concerning, particularly given the high prevalence of keratoconus in Saudi Arabia and the importance of early detection and intervention in preventing disease progression.

The overall low awareness of keratoconus observed in this study, with 414 participants (82.9%) demonstrating poor awareness, is consistent with other studies conducted in Saudi Arabia. For example, Kordi (2022) reported that 94.1% of participants in Medina had poor awareness of keratoconus, while Alamri (2023) found similarly low levels in Aseer, where only 14.26% of participants demonstrated good awareness [8,11]. In a more recent study by AlSomali (2024) in the Eastern Province, only 26% of participants had a high level of awareness [10]. These findings suggest that public knowledge about keratoconus is insufficient across different regions of Saudi Arabia, despite its relatively high prevalence. Moreover, it highlights the urgent need for public health initiatives to improve awareness, particularly regarding the early signs, risk factors, and treatment options for keratoconus.

Although 286 participants (57.3%) in our study had heard of keratoconus, only 83 participants (16.6%) correctly identified it as a disorder of corneal thinning. This discrepancy between general awareness and specific knowledge mirrors the findings from Kordi’s (2022) study, where 39.8% of participants had heard of keratoconus, but only 14.9% could correctly define it [11]. Similarly, in AlSomali’s (2024) study, 27.1% of participants correctly identified keratoconus as corneal thinning, highlighting a common pattern of insufficient detailed knowledge about the condition across different regions [10]. These findings emphasize that mere exposure to the term "keratoconus" is not enough to foster understanding; targeted educational efforts are required to bridge this knowledge gap and encourage early diagnosis [12,13].

The association between keratoconus and allergies is well-established, and frequent eye rubbing, often due to allergies, is a significant risk factor for the progression of keratoconus [14-16]. In this study, 248 participants (49.7%) reported a history of allergies, with 151 participants (54.3%) having eye allergies. The prevalence of allergies, especially ocular allergies, is consistent with findings from similar studies, such as those by Kordi (2022) and Alamri (2023), who reported 34.9% and 41.6% of participants with allergies, respectively [8,11]. The widespread prevalence of allergies, combined with the high incidence of eye rubbing, underscores the importance of educating the public about the risks of this behavior, particularly in relation to keratoconus.

In our study, 52.7% of participants believed that frequent eye rubbing could lead to keratoconus, which aligns with AlSomali's (2024) finding that 42.3% of participants recognized this association [10]. However, a significant proportion of participants in our study (37.3%) were uncertain about this link, indicating that knowledge about the harmful effects of eye rubbing remains incomplete. A significant body of research has established a strong association between keratoconus and the habit of eye rubbing, which is often a response to ocular irritation or fatigue [14]. Eye rubbing has been identified as a critical risk factor for the development and progression of keratoconus, with studies showing that 66% to 73% of keratoconus patients reported a history of frequent eye rubbing, and up to 80% of keratoconus patients reported this behavior compared to 58% in normal controls [17,18]. Mechanistically, eye rubbing can cause mechanical trauma to the cornea, leading to keratocyte damage, inflammatory responses, and the activation of cytokines and proteases, which contribute to corneal weakening and ectasia [19]. This risk is exacerbated in individuals with atopic conditions, who are more prone to ocular irritation and frequent eye rubbing [20]. Various studies, including multivariate analysis, have confirmed eye rubbing as the only statistically significant risk factor for keratoconus after adjusting for other variables, such as atopy [20]. These findings suggest that public health efforts should specifically target misconceptions and lack of awareness about the risks of eye rubbing, particularly among those with allergic conditions.

Our findings did not reveal significant associations between demographic factors such as gender, age, or education level and keratoconus awareness. This contrasts with findings from other studies in Saudi Arabia. Alamri (2023) reported that younger participants and those with higher education demonstrated significantly better awareness of keratoconus [8]. Similarly, AlSomali (2024) found that younger participants, singles, and those with higher education levels had better awareness [10]. These discrepancies could be due to regional differences in access to education and healthcare resources or varying levels of exposure to public health campaigns.

However, our study did find that participants with a history of refractive errors or other eye conditions, as well as those with a family history of keratoconus, had significantly better awareness of the disease. This finding is consistent with previous studies, such as Kordi (2022) and AlSomali (2024), both of which found that individuals with visual problems or a family history of keratoconus were more likely to have good awareness [10,11]. These groups are likely more engaged with eye care services and thus more informed about keratoconus. This highlights the need for targeted awareness campaigns among the general population, particularly those without known risk factors or a history of eye conditions.

The imperative need for public health interventions is underscored by the high prevalence of keratoconus in Saudi Arabia and the low awareness levels observed in Taif and other regions. The primary objective of educational campaigns should be to increase awareness of keratoconus, with a particular emphasis on its early symptoms, risk factors, and the significance of early diagnosis. Additionally, it is imperative to inform the public about the hazards of eye irritation, particularly for those with allergies. Healthcare professionals in ophthalmology and primary care are essential in raising awareness and should consider conducting routine screenings for at-risk individuals. The public's comprehension and early detection of keratoconus could be significantly enhanced through the utilization of digital tools and social media.

Limitations

This study has several limitations that should be acknowledged. First, the cross-sectional design limits the ability to establish causality between awareness levels and actual behavior related to keratoconus prevention. Second, the reliance on self-reported data may introduce bias, particularly in relation to behaviors such as eye rubbing, which may be underreported. Third, the study population, while representative of Taif city, may not fully reflect the broader Saudi population, limiting the generalizability of the findings. Future studies could address these limitations by employing longitudinal designs, objective measures of eye rubbing, and larger, more diverse samples.

## Conclusions

This study demonstrates a low level of awareness about keratoconus in Taif, Saudi Arabia, despite the high prevalence of the disease in the region. These findings are consistent with similar studies conducted across Saudi Arabia and highlight the need for comprehensive public health strategies to raise awareness, promote early detection, and reduce the risks associated with keratoconus, particularly through targeted education about modifiable behaviors such as eye rubbing. Effective public health interventions could significantly improve disease outcomes and reduce the burden of keratoconus on individuals and the healthcare system.
